# Mitochondria-derived peptide MOTS-c: effects and mechanisms related to stress, metabolism and aging

**DOI:** 10.1186/s12967-023-03885-2

**Published:** 2023-01-20

**Authors:** Wei Wan, Lieliang Zhang, Yue Lin, Xiuqing Rao, Xifeng Wang, Fuzhou Hua, Jun Ying

**Affiliations:** 1 Department of Anesthesiology, The Second Affiliated Hospital of Nanchang University, Nanchang, 330006 Jiangxi China; 2 Department of Anesthesiology, The First Affiliated Hospital of Nanchang University, Nanchang, 330006 Jiangxi China; 3 Key Laboratory of Anesthesiology of Jiangxi Province, 1# Minde Road, Nanchang, 330006 Jiangxi People’s Republic of China

**Keywords:** MOTS-c, Mitochondrial gene, Stress response, Aging, Inflammation

## Abstract

MOTS-c is a peptide encoded by the short open reading frame of the mitochondrial 12S rRNA gene. It is significantly expressed in response to stress or exercise and translocated to the nucleus, where it regulates the expression of stress adaptation-related genes with antioxidant response elements (ARE). MOTS-c mainly acts through the Folate-AICAR-AMPK pathway, thereby influencing energy metabolism, insulin resistance, inflammatory response, exercise, aging and aging-related pathologies. Because of the potential role of MOTS-c in maintaining energy and stress homeostasis to promote healthy aging, especially in view of the increasing aging of the global population, it is highly pertinent to summarize the relevant studies. This review summarizes the retrograde signaling of MOTS-c toward the nucleus, the regulation of energy metabolism, stress homeostasis, and aging-related pathological processes, as well as the underlying molecular mechanisms.

## Introduction

Mitochondria have long been known as the energy-producing structures of the cell and the main site of aerobic respiration. As the descendants of prokaryotic endosymbionts, mitochondria have their own genetic material and genetic system. In addition to supplying energy to the cell, mitochondria are also involved in cellular stress responses, information transfer, cell death, and aging processes [1]. Since mitochondria regulate a wide range of functions and processes in the cell, there is intricate coordination of mitochondrial signaling in multiple networks. As a consequence, mitochondrial dysfunction can lead to a variety of pathologies and diseases such as oxidative stress, metabolic imbalance, inflammatory response, neurodegenerative diseases and aging [2–6]. Overall, mitochondrial and age-related diseases represent an enormous and growing global socioeconomic burden. Therefore, understanding the mechanisms through which mitochondria affect these diseases is crucial for improving the quality of life of the elderly and promoting healthy aging.

In 2015, the MOTS-c peptide (mitochondrial open reading frame of the 12S rRNA type-c), encoded by mitochondrial DNA, was discovered [7]. MOTS-c is mainly activated by stress and exercise, while its expression decreases with aging. MOTS-c was initially found to regulate glucose uptake, lipid metabolism and insulin resistance, as well as being involved in aging-related physiological and pathological changes [7]. With the increasing number of relevant studies in recent years, MOTS-c has gradually been found to also affect obesity, inflammation, neuroprotection and aging-induced hypokinesia, potentially contributing to healthy aging [8–11]. A systematic review of MOTS-c is necessary because of the powerful potential functions it exhibits and the increasing number of related studies.

This review summarizes retrograde MOTS-c signaling toward the nucleus, its role in multiple physiological and pathological processes, as well as the underlying molecular mechanisms.

### Mitochondrial DNA

Mitochondria originate from alpha-proteobacteria engulfed by ancestors of eukaryotic cells [12]. Mitochondria have a variety of interrelated functions, the best known being the production of ATP through oxidative phosphorylation (OXPHOS), as well as the production of many biosynthetic intermediates and participation in cellular stress responses [1]. Human mitochondrial DNA (mtDNA) is a double-stranded DNA (dsDNA) molecule of 16.6 kb, which encodes 11 mRNAs (translated into 13 proteins), 2 rRNAs (12S and 16S rRNA), and 22 tRNAs [13]. All 13 mtDNA-encoded proteins constitute a minority of the OXPHOS subunits, but they are nevertheless essential [14]. Although the mitochondrial genome was thought to be relatively simple, encoding just 13 proteins dedicated to energy production, it was later found to be a highly complex system with numerous previously unknown features and functions of encoded peptides.

The regulation of mtDNA expression is highly complex and involves the control of many processes, such as maintenance and transcription of mtDNA, mitochondrial RNA processing, mitochondrial mRNA stability and polyadenylation, mitochondrial tRNA modification, and translation by mitochondrial ribosomes [15]. In addition, analysis of the genome, transcriptome and proteome revealed the presence of a large number of unannotated short open reading frames (sORF or smORF), which may be translated into peptides and proteins [16]. Many studies have suggested that some mRNAs contain multiple ORFs, with a shorter ORF in the *5′UTR* of the longer downstream ORFs [17, 18]. These short ORFs are named upstream ORFs (uORFs). Initially, uORFs were not thought to encode proteins and were considered to function primarily as cis-acting elements, mediating ribosomal scanning and translation of longer downstream ORFs [17, 18]. Subsequent studies have shown that at least some uORFs are translated into peptides and play a key role in the translation of downstream long ORFs [19]. Some sORFs have already been shown to have biological activities in metabolism, apoptosis and development [20, 21]. The discovery of new sORFs encoded by nuclear and mitochondrial DNA has greatly enriched the knowledge of the human genome.

### Mitochondria-derived peptides

**Fig. 1:**
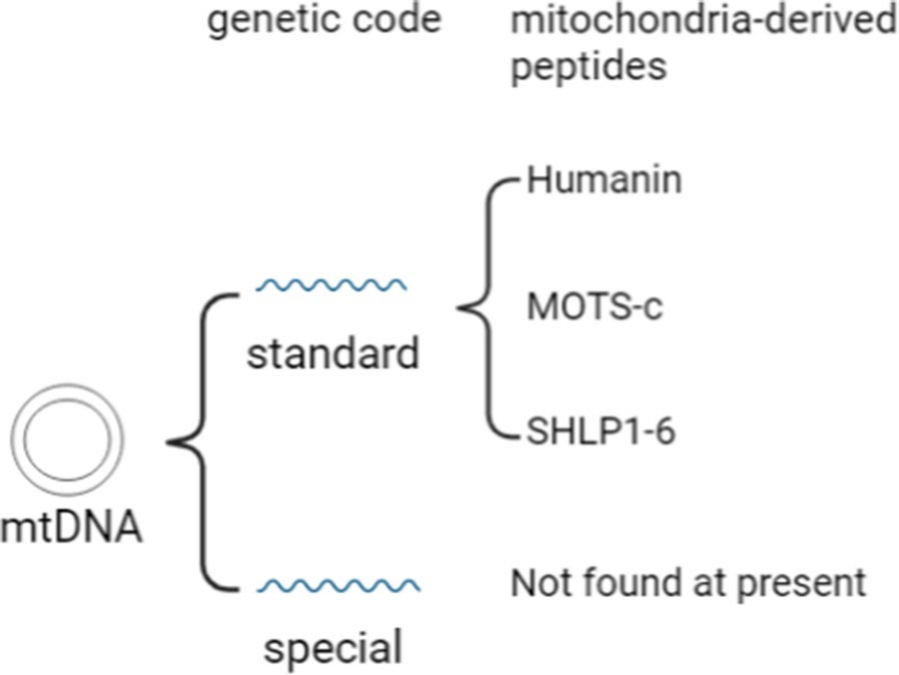
Mitochondria-derived peptides encoded by mtDNA. Mitochondrial DNA has two sets of genetic code, standard and specific. The mitochondria-derived peptides (Humanin, MOTS-c and SHLP1-6) found so far are all encoded by the standard genetic code

Mitochondrial DNA has a special genetic code, in which *ATA* and *ATT* are used as start codons in addition to the standard start codon *ATG*, while *AGA* and *AGG* are used as stop codons instead of coding for arginine, and the standard stop codon *UGA* is used to code for tryptophan [22, 23]. Mitochondrial DNA (heavy and light strand) can be classified into four categories of sORFs of 9–40 amino acids based on the standard and special genetic codes [24]. No mitochondria-derived peptide (MDPs) was found in the mitochondrial DNA using the special genetic codes. Thus, the MDPs identified to date were encoded by the standard genetic code. These include Humanin [20, 25] and SHLP1-6 (Small Humanin-Like Peptide 1–6) [26], encoded by the 16S rRNA, as well as MOTS-c (Mitochondrial Open reading frame of the Twelve S rRNA type-c) [7], encoded by the 12S rRNA (Fig. 1). Humanin, MOTS-c and SHLP6 were encoded using the standard genetic code heavy chain class, and SHLP2-5 were encoded using the standard genetic code light chain class [24].

The first discovered biologically active MDP was Humanin, which has anti-apoptotic and neuroprotective effects [20, 25]. In 2001, Hashimoto et al. discovered Humanin for the first time using a cDNA library extracted from tissue from healthy parts of the brains of Alzheimer's disease patients. Humanin is encoded by a 75 bp ORF that is translated into peptides of 21 or 24 amino acids depending on the location of the translation machinery [27]. Humanin performs multiple functions by binding to intracellular molecules and cell membrane receptors (such as Aβ17–28, FPRL-1/2, CNTF, IGFBP-3, Bax and tBid) [27]. Humanin can enhance cellular resistance to Alzheimer's disease-associated toxins such as β-amyloid, improve insulin sensitivity, prevent oxidative stress damage due to ischemia/reperfusion (I/R), hypoxia, or starvation, and increase the resistance to apoptosis [27, 28]. The discovery of Humanin opened the field of MDP research and has contributed to the discovery of further MDPs, providing new potential targets for the treatment of many age-related diseases.

Subsequently, Cobb et al. searched for potential sORFs encoding short peptides in 16S rRNA and identified six sequences encoding 20–38 amino-acid-peptides, which were named SHLP1-6 [26]. In mouse β-cells (NIT-1) and human prostate cancer cells (22Rv1), SHLP2 and SHLP3 enhanced cell viability and reduced apoptosis. SHLP2 and SHLP4 promoted cell proliferation in NIT-1β cells, while SHLP6 significantly increased apoptosis in NIT-1 and 22Rv1 cells [26]. Interestingly, treatment of age-related macular degeneration (AMD) with SHLP2 restored normal levels of OXPHOS complex protein subunits, increased the mitochondrial DNA copy number, attenuated amyloid beta-induced cellular and mitochondrial toxicity, inducesd anti-apoptotic effects, and prevented the loss of viable cells and mitochondria [29].

As early as 1983, cDNAs corresponding to the 12S rRNA region have been cloned, but the exact sequence had not been determined at the time [30]. Subsequently, an electronic search for potential sORFs in 12S rRNA revealed that one of the sequences consisted of 51 base pairs, and the sequence was translated into a 16-amino acid peptide named MOTS-c (mitochondrial open reading frame of the 12S rRNA type-c) [7]. Moreover, basic local alignment search tool (BLAST) searches against the Human Expressed Sequence Tag (EST) database indicated a mitochondrial DNA origin for MOTS-c and ruled out a nuclear origin [7]. Based on the differences between the nuclear and mitochondrial genetic codes, MOTS-c translation is most likely to occur in the cytoplasm. Human mitochondria use ‘‘*AGA*’’ and ‘‘*AGG*’’ as stop codons, and mitochondrial translation of MOTS-c would result in tandem start and stop codons [7, 31]. By contrast, this codon would encode arginine in the traditional cytoplasmic translation. However, the mechanism of mitochondrial export of MOTS-c transcripts remains unknown. MOTS-c is mainly present in skeletal muscle and blood, while its concentration decreases with age [7, 11]. MOTS-c has significant effects on stress responses, cellular metabolism, sports ability, and inflammation through altered expression of nuclear genes (details described below) [7, 8, 32–34].

### MOTS-c mediates the communication of mitochondrial genes with nuclear genes

**Fig. 2:**
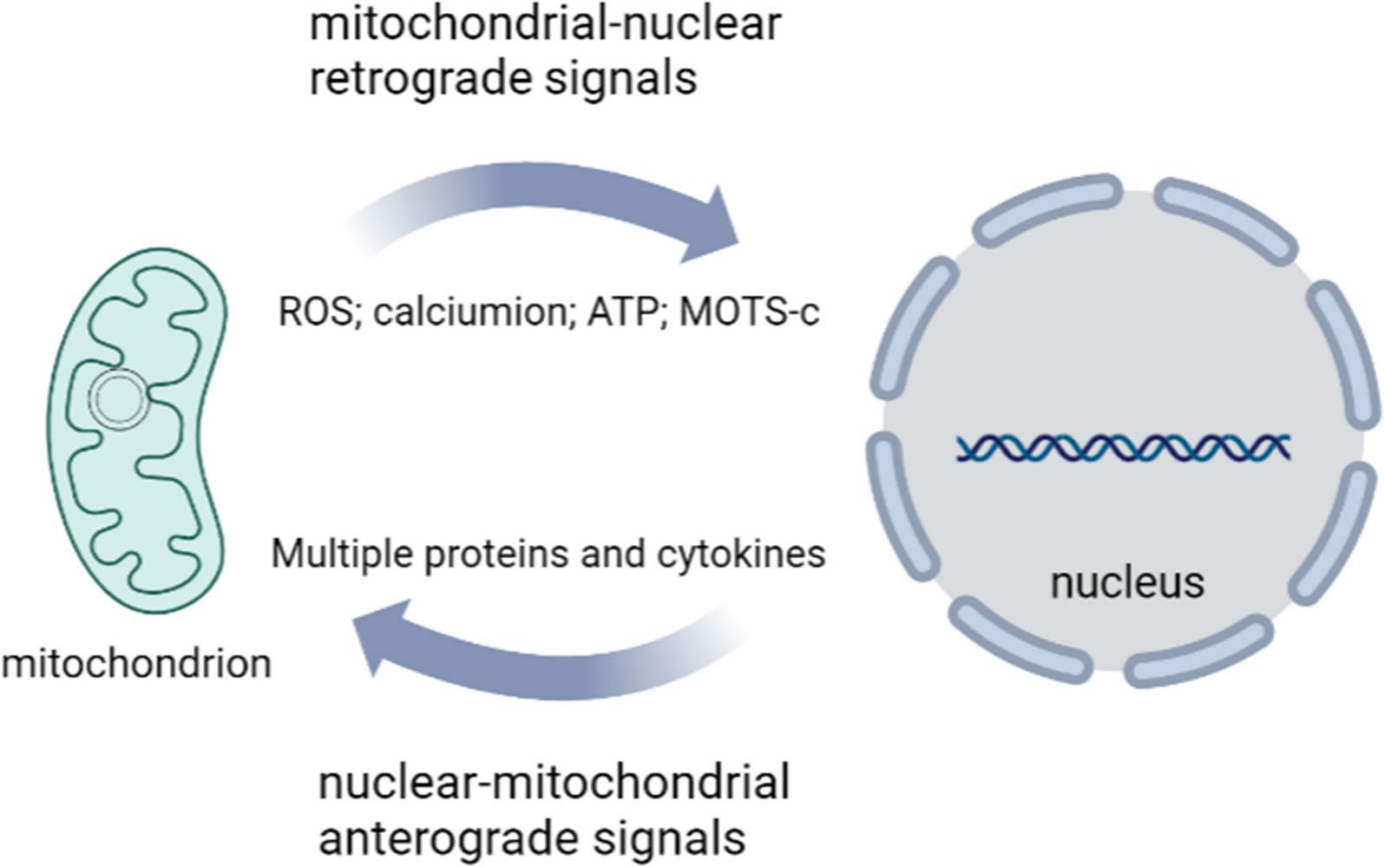
Communication of mitochondrial genes with nuclear genes. The nucleus sends prograde signals through the expression of proteins and cytokines that regulate mitochondrial function. Mitochondria can also send retrograde signals through ROS, calciumion, ATP and MOTS-c to regulate nuclear gene expression

As “energy factories” and organelles that regulate metabolism, mitochondria contain more than 1000 proteins encoded in the nuclear genome and are regulated by a variety of nuclear signals. Traditionally, the mitochondrial genome was thought to encode only 13 proteins as structural components of the electron transport chain that play key roles in oxidative phosphorylation [13]. To coordinate the multiple functions of mitochondria, they undergo extensive communication with the nucleus, including mostly nuclear-mitochondrial anterograde signals, and few mitochondrial-nuclear retrograde [35]. The currently known retrograde signaling is mainly activated by OXPHOS and mitochondrial DNA defects, which activate AMPK (AMP-activated protein kinase) or NF-κB (Nuclear factor-κB) pathways to alter gene expression in the nucleus through mediators such as ROS, calcium ion concentration and ATP content [35]. However, MOTS-c can be translocated to the nucleus in response to metabolic stress and regulate adaptive nuclear gene expression [33, 36–38] (Fig. 2). This discovery complements the mutual signaling communication between mitochondrial and nuclear genes and reveals a complex regulatory mechanism of mitochondrial function.

**Fig. 3:**
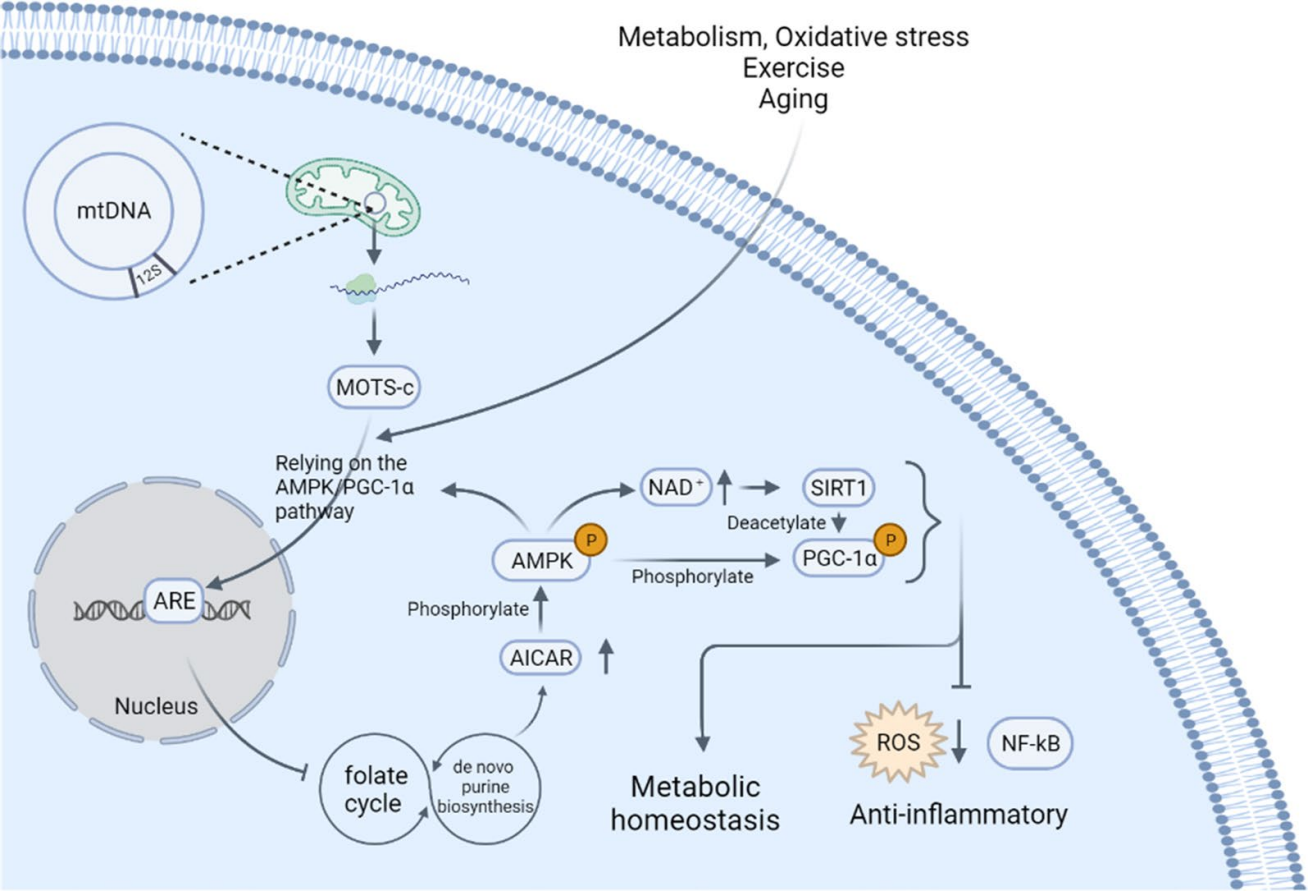
Mechanism and function of MOTS-c. Short open reading frame on 12S rRNA of mitochondrial DNA transcribes mRNA in mitochondria. Subsequently, the mRNA translocates to the cytoplasm to translate MTOS-c. MOTS-c is triggered to translocate into the nucleus by stress, exercise and aging through the AMPK/PGC-1α-dependent pathway, thereby regulating the expression of genes with antioxidant response elements (ARE) and stress adaptation-related. MOTS-c can mainly regulate the folate cycle and de novo purine biosynthesis pathway, leading to an increase in AICAR, which phosphorylates and activates AMPK. AMPK can regulate energy homeostasis and produce anti-inflammatory effects by activating SIRT1 and PGC-1α (Phosphorylation and deacetylation). In addition, AMPK can promote nuclear translocation of MOTS-c, forming a feedback loop

In the resting state, MOTS-c exhibits a predominantly extranuclear localization pattern, but when cells were subjected to metabolic stress (glucose restriction, serum deprivation, or oxidative stress), MOTS-c was found to rapidly translocate into the nucleus as early as 30 min after stress induction, and return to its basic extranuclear state within 24 h [36]. Moreover, MOTS-c translocates to the nucleus in an AMPK-dependent manner, indicating that pharmacological and genetic interventions that inhibit AMPK activity may prevent stress-induced nuclear translocation of MOTS-c. Activation of AMPK by metformin and 5-aminomidazole-4-carboxamide ribonucleotide (AICAR) mimics stress-like cellular responses and leads to the translocation of MOTS-c to the nucleus (Fig. 3) [36]. ROS may be another potential stress metabolite affecting translocation, as N-acetylcysteine treatment inhibits oxidant-induced nuclear translocation [33, 39]. Substitution of a hydrophobic core residue of MOTS-c (8YIFY11, which interacts with other proteins) prevents its entry into the nucleus, suggesting that nuclear translocation of MOTS-c may require interaction with other proteins. Furthermore, increasing the concentration of MOTS-c alone does not trigger its nuclear translocation [36].

Upon entry into the nucleus, MOTS-c interacts with a variety of stress response transcription factors, including nuclear factor red lineage 2-related factor 2 (NFE2L2/NRF2), as well as activating transcription factors 1 and 7 (ATF1/ATF7) [38]. Importantly, MOTS-c can bind to the promoter regions of NRF2 target genes with antioxidant response element (ARE) sequences. Activated NRF2 regulates drug metabolism, antioxidant defenses and oxidative signaling by mediating the expression of enzymes and signaling proteins [40]. In addition, some transcription factor binding motifs are enriched in the promoters of genes regulated by MOTS-c genes, such as ATF1/ATF7 and JUND (also known as JunD proto-oncogene) [38]. MOTS-c plays roles in obesity, insulin resistance, exercise, inflammation and lifespan through retrograde signaling affecting nuclear gene expression (such as the AMPK pathway and pro-inflammatory factors).

### The pathways regulated by MOTS-c

#### AMPK

AMP-activated protein kinase (AMPK) is a highly conserved heterotrimeric kinase complex consisting of a catalytic (α) subunit and two regulatory (β, γ) subunits. AMPK is activated under energy stress conditions such as nutrient deficiency or hypoxia, as well low intracellular ATP with concomitant increase of AMP [41]. In their first discovery of MOTS-c, Lee and colleagues identified its target of action as the folate-methionine cycle and the directly tethered de novo purine biosynthesis pathway through global unbiased metabolomic profiling [7]. The levels of 5-methyltetrahydrofolate (5Me THF) and methionine both decreased after MOTS-c treatment, while homocysteine levels increased. However, the 5Me THF levels decreased first, which suggests that regulation of the folate cycle occurred earlier [7]. Subsequently, the level of endogenous AICAR (5-aminoimidazole-4-carboxamide ribonucleotide) in cells was elevated due to the blockage of de novo purine biosynthesis. It is known that AICAR, similar to metformin, is an activator of AMPK. AICAR is phosphorylated by cellular kinases to form ZMP, which acts as an AMP mimetic that directly binds and activates AMPK [42, 43].

MOTS-c treatment induced AMPKα and Akt phosphorylation in a time- and dose-dependent manner, causing cells to display a significantly enhanced glycolytic response. Knockdown of AMPKα2 or AMPKα1/2 in cells using siRNA and the use of AMPK inhibitor compound C reduced the increase of glycolysis by 16%, 30% and 40%, respectively. This suggests that AMPK activation plays a partial role in mediating the action of MOTS-c. This is also suggested by the reversal of the enhanced glycolytic response by supplementation of folic acid in the culture medium [7]. Interestingly, AMPK is also necessary for MOTS-c stress-responsive nuclear translocation. Subcellular isolation and immunofluorescence imaging showed that pharmacological and genetic interventions that inhibit AMPK activity prevent the stress-induced nuclear translocation of MOTS-c, which rapidly enters the nucleus within 1 h after metformin and AICAR treatment [36]. This suggests that AMPK both promotes the nuclear translocation of MOTS-c and mediates its physiological effects, forming a positive feedback loop.

As a key downstream molecule of MOTS-c, AMPK mediates a variety of effects such as metabolic homeostasis, insulin resistance, fat accumulation, exercise, inflammation, osteoporosis, cardiovascular protection, and aging [7, 9, 44–49].

#### SIRT1/PGC-1α

Sirtuin1 (SIRT1) is an evolutionarily conserved NAD^+^-dependent deacetylase involved in a variety of cellular metabolic and aging processes through deacetylation of target proteins [50]. PGC-1α, a member of the transcriptional coactivator family, regulates mitochondrial biogenesis and respiratory function through the regulation of the energy sensors AMPK and SIRT1 [51]. Since SIRT1 can mediate the activation of AMPK by NAD^+^, Lee and colleagues also tested the possible role of SIRT1 in mediating the effect of MOTS-c on glycolysis [7]. Pharmacological or genetic inhibition of SIRT1 activation led to a significant reduction in the rate of glucose-stimulated glycolysis [7]. This suggests that SIRT1 is partially required for certain effects of MOTS-c.

Subsequent studies revealed that PGC-1α, a gene involved in the co-activation of mitochondrial biogenesis and metabolic processes, was significantly upregulated by MOTS-c, suggesting its functional involvement [52]. MOTS-c inhibits ROS production by activating PGC-1α, resulting in an anti-inflammatory effect (decreases levels of pro-inflammatory cytokines TNF-α, IL-1β and IL-6, while increasing the levels of anti-inflammatory cytokine IL-10). PGC-1α knockdown reversed the inhibitory effect of MOTS-c on ROS production while increasing the levels of TNF-α, IL-1β and IL-6, as well as reducing IL-10 levels [52].

MOTS-c promotes the expression of PGC-1α through a mechanism that is probably mediated by AMPK. Previous studies have shown that AMPK activates PGC-1α via SIRT1, which causes deacetylation of PGC-1α [53, 54]. It has also been suggested that AMPK directly activates PGC-1α by phosphorylating PGC-1α [55]. In any case, AMPK plays an important role in the activation and expression of PGC-1α. MOTS-c treatment promoted AMPK phosphorylation and PGC-1α expression, while inhibition of AMPK activation with compound C or a specific siRNA suppressed PGC-1α expression, as shown by western blot analysis [52]. This suggests that MOTS-c exerts anti-inflammatory effects through the AMPK-PGC-1α-ROS axis. Another study also demonstrated that SIRT1 overexpression or SIRT1 activator (SRT1720) treatment increased the protein and mRNA expression of PGC-1α [56]. Interestingly, the SIRT1-PGC-1α pathway also mediates the production and/or secretion of MOTS-c in skeletal muscles [56].

SIRT1/PGC-1α also plays a key role in the increase of MOTS-c expression due to exercise [56–58]. PGC-1α knockdown downregulated MOTS-c protein and mRNA expression in C2C12 myotubes, whereas PGC-1α overexpression upregulated both the protein and mRNA expression of MOTS-c in C2C12 myotubes [57]. In addition, mice subjected to an exercise regimen showed significantly increased protein and mRNA levels of MOTS-c in the plasma and skeletal muscle, as well as protein expression of SIRT1 and PGC-1α compared to mice that did not receive exercise intervention [56]. Importantly, there appears to be a MOTS-c-PGC-1α feedback loop in skeletal muscle in which MOTS-c regulates PGC-1α expression, while PGC-1α expression regulates the levels of MOTS-c in muscles and plasma [57, 58]. However, PGC-1α is a transcriptional coactivator that does not bind directly to DNA but regulates gene expression by interacting with other transcription factors. Therefore, the direct upstream factors regulating MOTS-c expression remain to be elucidated.

#### NF-κB

Nuclear factor-κB (NF-κB) plays crucial roles in the immune system, regulating the expression of inducers and effectors at many points in the broad network of the immune response [59, 60]. As stated in the introduction, MOTS-c inhibits ROS production via the AMPK-PGC-1α axis. ROS may act as a secondary messenger to activate NF-κB, and MOTS-c may inhibit NF-κB by reducing ROS production, thereby decreasing the levels of pro-inflammatory cytokines TNF-α, IL-1β and IL-6, while increasing the levels of anti-inflammatory cytokine IL-10 [9, 52]. In a study on osteolysis, UHMWPE particles were found to significantly activate NF-κB and STAT1 (signal transducer and activator of transcription [1], inducing the nuclear translocation of NF-κB as evidenced by western blot analysis and immunofluorescence. MOTS-c blocked the increase of NF-κB and STAT1 levels in a dose-dependent manner and inhibited the nuclear translocation of NF-κB [52]. Further detection of JAK1 and IFN-γ, the upstream factors of STAT1, revealed that JAK1 levels were reduced, while both the mRNA and protein levels of IFN-γ were significantly downregulated by MOTS-c treatment. However, the classical upstream molecules of NF-κB, ERK1/2 and p38 MAPK (mitogen activated protein kinase), did not exhibit significant differences [52]. However, another study concluded that MOTS-c inhibited MAPK phosphorylation in macrophages while enhancing the expression of aromatic hydrocarbon receptor (AHR) and STAT3 [61]. In sepsis caused by methicillin-resistant *S. aureus* (MRSA), the three major kinases of the MAPK pathway, ERK1/2, p38 and JNK, are activated, while MOTS-c treatment reduced the phosphorylation levels of all three kinases. At the same time, MOTS-c promoted AHR expression and STAT3 phosphorylation, the two major negative regulators of pro-inflammatory signaling [61]. In addition, AHR inhibitors reduced the inhibitory effect of MOTS-c on three MAPK molecules, suggesting that MOTS-c exerts its anti-inflammatory effect in an AHR-dependent manner [61]. It was also suggested that LPS stimulation induced a significant increase of AMPK phosphorylation in lung tissue, leading to the activation of ERK, JNK and P38 MAPK, while MOTS-c treatment significantly reduced the phosphorylation levels of ERK and JNK MAPK [9, 45]. Overall, whether MOTS-c activates NF-κB via the MAPK pathway requires further study.

### Multiple functions of MOTS-c

#### MOTS-c improves metabolic homeostasis and ameliorates insulin resistance

**Fig. 4:**
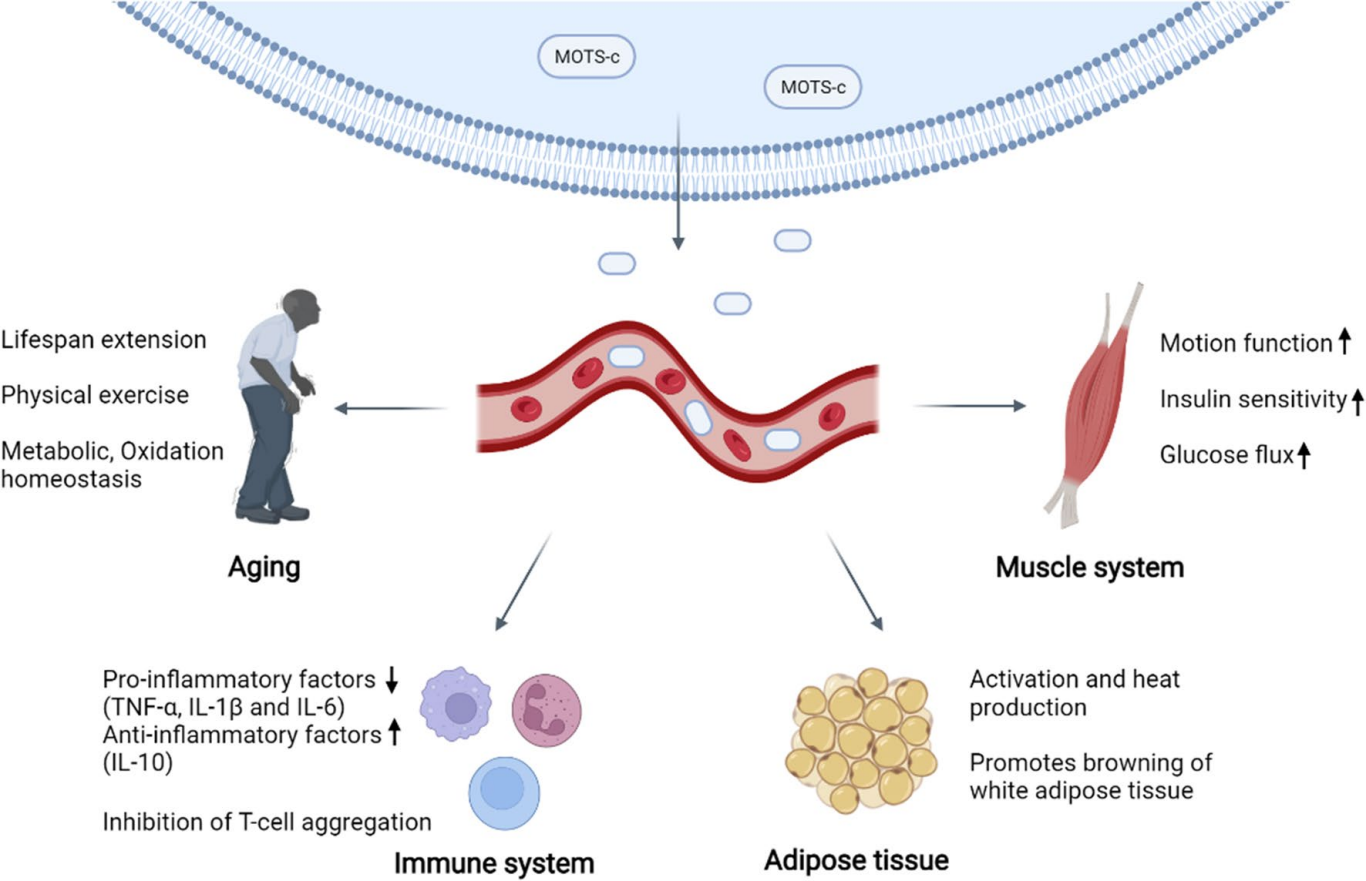
The system role of MOTS-c. MOTS-c acts on multiple systems such as muscle, adipose tissue and immune system through blood circulation. MOTS-c can increase muscle's motor function and insulin sensitivity, promote the conversion from white adipose tissue to brown adipose tissue while activating brown adipose tissue, additionally decreasing the expression of inflammatory factors and increasing the anti-inflammatory effect. Besides, MOTS-c is beneficial for antioxidant stress, metabolic homeostasis, exercise capacity and healthy aging

Initial studies on MOTS-c found modest reductions in body weight, food intake and blood glucose levels in MOTS-c-treated high fat diet (HFD)-fed mice [7]. MOTS-c treatment enhanced cellular glucose flux in vitro and reduced glucose levels in mice fed a normal diet. Significantly enhanced glucose clearance in the glucose tolerance test and hyperinsulin-orthoglycemic clamping studies demonstrated improved systemic insulin sensitivity [7]. In addition, enhanced skeletal muscle-specific insulin sensitivity was demonstrated by deuterated glucose injection during clamping [7]. Interestingly, the muscles of aged mice were more insulin resistant than those of young mice, but MOTS-c treatment restored the sensitivity of aged mice to a level comparable to that of young mice. While MOTS-c treatment had no effect on the body weight of mice fed a normal diet, when administered to mice fed a HFD, it significantly decreased the rate of obesity and significantly reduced basal levels of circulating IL-6 and TNF-α associated with the pathogenesis of obesity and insulin resistance. In addition, MOTS-c treatment prevented HFD-induced hyperinsulinemia, indicating improved glucose homeostasis [7]. Overall, MOTS-c prevented HFD-induced obesity through increased energy expenditure, improved glucose utilization and insulin sensitivity (Fig. 4).

Subsequent studies investigated MOTS-c levels in physiological and pathological states to better understand the status and function of MOTS-c. However, MOTS-c levels in different states remain controversial. One study suggested that circulating MOTS-c levels are reduced in obese male children and adolescents, which is associated with insulin resistance, but not in obese female children and adolescents [62]. However, another study concluded that MOTS-c concentrations were increased in the blood of mothers and newborns in the obese group compared to healthy controls, and that concentrations of this peptide were correspondingly lower in mothers and newborns in the hypothyroid group compared to the obese group [63]. Increased MOTS-c levels in the blood of obese mothers imply that the body is trying to maintain high glucose utilization and limit the growth of adipose tissue, and may also be a factor in preventing islet damage [8]. In the absence of type 2 diabetes, the components of the metabolic syndrome (MS), especially liver fat, were positively associated with MOTS-c levels [64]. However, MOT-c expression was low in type 2 diabetes and correlated with glycated hemoglobin (HbA1c) [65]. This may indicate that endogenous concentrations of MOTS-c are elevated in the early stages of metabolic disease development in an effort to restore metabolic homeostasis. However, this compensation is diminished or disappears in the middle and late stages of metabolic disease. Chronic kidney disease also leads to mitochondrial dysfunction and reduced expression of MOTS-c [66]. In addition, decreased MOTS-c was an additional predictor of increased platelet reactivity to clopidogrel in patients with diabetes mellitus combined with coronary artery disease during 2 year follow-up [67].

Postmenopausal women are known to exhibit physiological changes, including weight gain, changes in the distribution of adipose tissue, and a decrease in insulin secretion and sensitivity [68]. MOTS-c treatment prevents postmenopausal obesity and insulin resistance [32, 44]. MOTS-c prevents weight gain and reduces adiposity in ovariectomized mice (used to mimic menopause) by reducing blood lipids and hepatic triacylglycerol levels, while also enhancing lipolysis [44]. Interestingly, this experiments also revealed that MOTS-c treatment significantly downregulated adipogenesis-related genes (*Fasn, Scd1*) and enhanced the expression of lipid oxidation-related genes. In addition, MOTS-c treatment also activated brown fat (described below) [44].

In type 2 diabetes (T2D) and obesity, three pathways of sphingolipid metabolism, monoacylglycerol metabolism and dicarboxylic acid metabolism are upregulated [69–71]. Importantly, MOTS-c improves insulin sensitivity, increases β-oxidation and prevents fat accumulation in mice by downregulating these pathways [72]. Specifically, ceramide is a byproduct of sphingolipid metabolism and is involved in the mechanism of insulin resistance. Sphingosine 1-phosphate (S1P) is also a byproduct of sphingolipid metabolism and is produced by the ceramide-sphingosine-S1P pathway [73, 74]. Elevated levels of ceramide and S1P were observed in T2D and obese subjects, and were reduced in MOTS-c-treated mice [72]. This suggests that MOTS-c may ameliorate insulin resistance by reducing the levels of sphingolipid metabolites. In the monoacylglycerol metabolic pathway, monoacylglycerol is hydrolyzed by membrane-bound lipoprotein lipase (LPL) and transported to tissues. LPL activity is inhibited by angiopoietin-like 4 (ANGPTL4) to regulate the transport of fatty acids and monoacylglycerols to tissues [75]. In mice treated with MOTS-c, increased levels of ANGPTL4 may inhibit LPL and prevent the accumulation of fat in muscles, thereby ameliorating insulin sensitivity [72]. Increased dicarboxylic acid (DCA) levels are generally considered to indicate dysregulated mitochondrial and peroxisomal β-oxidation. Decreased plasma DCA in mice treated with MOTS-c indicates a higher efficiency of normal β-oxidation [72]. In a recent study, MOTS-c treatment significantly alleviated hyperglycemia and improved insulin sensitivity in gestational diabetic mice, and reduced mortality in offspring [76]. In conclusion, there may be more ways for MOTS-c to regulate metabolic homeostasis and insulin resistance, and this is a promising area for future research.

#### MOTS-c regulates the activation of brown adipose tissue

Adipose tissue is broadly classified as brown, white and brown-like adipose tissue. Brown adipose tissue (BAT) is the main source of non-shivering thermogenic heat production during cold adaptation. BAT is highly vascularized, rich in mitochondria as well as multi-compartmental lipid droplets, and generates heat by burning lipids via uncoupling protein-1 (UCP1) [77, 78]. Recently, it has been suggested that MOTS-c activates BAT and increases white fat browning, thereby enhancing cold adaptation [79]. In acute cold exposure, MOTS-c treatment upregulated RNA levels of thermogenic genes (PGC-1α, UCP1 and Elovl3). HE staining showed that single-compartment intracellular lipid droplets of white fat became multi-compartmental lipid droplets and gradually developed a denser structure [79]. This suggests that MOTS-c increases the browning of white fat. In addition, western blot analysis showed that MOTS-c treatment continued to significantly enhance the expression of UCP1 during cold exposure, thereby activating BAT [79]. Importantly, MOTS-c treatment leads to the phosphorylation of ERK (a member of the MAPK family) without increasing the total amount of ERK protein. Pharmacological ERK inhibitors severely inhibited ERK phosphorylation induced by MOTS-c, reduced the increase of protein levels of PGC-1α and UCP1, while also reducing their mRNA expression [79]. This suggests that that MOTS-c activates BAT and causes white fat browning through the ERK pathway.

MOTS-c also activates BAT through regulation of mitochondria. MOTS-c treatment resulted in a significant upregulation of genes related to mitochondrial biogenesis (*PGC-1α, NRF1*), mitochondria-encoded genes (*MT-ND1, MT-CO1*) and OXPHOS-related genes, leading to the biogenesis of more mitochondria with abundant cristae, which is associated with increased respiratory capacity [44, 80, 81]. In addition to mitochondrial biogenesis, MOTS-c appears to have a role in other mitochondrial dynamics, but the related findings remain controversial. Some studies suggested that MOTS-c treatment resulted in significant upregulation of the mRNA levels of both mitochondrial fusion genes (*Mfn1 and Mfn2*), and longer tubular mitochondria were seen [80–82]. Elevated expression of mitophagy-related genes (*PINK1, PARK2* and *ATG7*) was also found. This suggests that MOTS-c can promote mitochondrial biogenesis, mitochondrial fusion and mitophagy processes. However, flow cytometry and fluorescence microscopy showed that MOTS-c treatment caused a concentration-dependent decrease in the number of mitochondria per cell [80]. This may be due to the fact that mitochondrial fusion and autophagy occur more drastically, or occur earlier. In addition, MOTS-c treatment promoted the stabilization of the internal environment of aged mesenchymal stem cells (MSCs) by reducing oxygen consumption and ROS production, thereby significantly enhancing intra-mitochondrial homeostasis [49]. In a different approach, cytoplasmic hybrid (cybrid cells) containing 3243 *A* to *G* mutant mitochondrial DNA were generated, which resulted in mitochondrial dysfunction. A recent study found that neither endogenous transfection with MOTS-c nor exogenous treatment showed significant effects on ATP content or mRNA and protein levels of the mitochondrial complex in mutant cybrid cells [83]. However, the specific process needs further research.

#### Anti-inflammatory and immune system effects of MOTS-c

Inflammation is an evolutionarily conserved protective mechanism that aims to maintain the stability of the body's internal environment in the face of infection or injury [84]. However, overproduction of pro-inflammatory factors and overreactive immune responses may lead to multi-organ dysfunction and tissue damage [85]. As described above, MOTS-c can activate several molecules such as AMPK, SIRT1 and NF-κB, while also inhibiting ROS production. This suggests a potential anti-inflammatory effect of MOTS-c, which is also consistent with a large number of later studies. For example, in sepsis caused by methicillin-resistant *Staphylococcus aureus* (MRSA), MOTS-c significantly increased the survival and reduced the bacterial load in mice while decreasing the levels of pro-inflammatory cytokines (TNF-α, IL-6, IL-1β) as well as increasing the levels of the anti-inflammatory cytokine IL-10 [61]. Similarly, in an inflammatory injury pain model, MOTS-c significantly inhibited formalin-induced ERK, JNK, and P38 activation as well as c-Fos expression (recognized as an important mediator of inflammatory pain), in addition to decreasing the levels of TNF-α, IL-6, and IL-1β [9]. This suggests that MOTS-c can exert anti-inflammatory effects by inhibiting the MAPK-c-Fos signaling pathway and reducing painful stimulation due to inflammation. In addition, MOTS-c was also found to have the same effects in an acute lung injury model, reducing pulmonary edema and inhibiting lung tissue neutrophil infiltration through downregulation of cytokine-induced neutrophil chemotactic factor-1 (CINC-1) and intercellular adhesion molecule-1 (ICAM-1) expression in lung tissue [45].

In addition to regulating the expression of pro-inflammatory factors, MOTS-c can also exert anti-inflammatory effects by targeting immune cells (such as T cells and macrophages) [8, 61]. A recent study showed that MOTS-c treatment reduced islet-infiltrating T cells and prevented the destruction of islet β cells, thereby slowing the progression of non-obese diabetes (NOD) [8]. Specifically, MOTS-c promoted the differentiation of CD4^+^CD25^+^FOXP3^+^ regulatory T cells (Tregs), which exhibited low glycolytic activity and showed therapeutic potential in type 1 diabetes (T1D) and other autoimmune diseases, through direct inhibition of mTOR complex 1 (mTORC1) signaling in T cells [86]. Conversely, MOTS-c inhibited the differentiation of CD4^+^ interferon gamma (IFNγ)^+^ T helper type 1 (Th1) cells, which have high glycolytic activity and are associated with the pathogenesis of T1D [87]. NOD mice treated with MOTS-c showed a decreased abundance of Th1 cells and an increase in the abundance of Treg cells in the spleen and pancreas, which significantly delayed disease progression [8]. According to previous studies, AMPK, an upstream molecule of mTOR, likely mediates the regulation of T cells by MOTS-c [41, 88]. Interestingly, IL-10, a potent anti-inflammatory cytokine that can prevent T1D, may also play a key role [89].

MOTS-c did not increase the number of macrophages in uninfected mice, but it increased the phagocytic and bactericidal capacity [61]. Osteoblasts are multinucleated giant cells formed by the fusion of mononuclear macrophages differentiated from myeloid progenitor cells in the bone marrow. MOTS-c inhibited the differentiation of bone marrow macrophages (BMMs) to mature multinucleated osteoblasts in a dose-dependent manner and did not affect the differentiation of osteoblasts. However, the exact mechanism is not clear and may be related to an increased osteoprotegerin (OPG)/nuclear factor-κB ligand (RANKL) receptor activator ratio in osteoblasts or it may be mediated by the AMPK pathway [48, 52]. This allowed MOTS-c treatment to significantly alleviate bone loss and slow the progression of osteoporosis. In agreement with these preclinical studies, postmenopausal women are at increased risk of obesity, insulin resistance, osteoporosis, cardiovascular disease, and cognitive decline [90]. Currently, the main treatment for postmenopausal pathologies is hormone therapy, but its risks and benefits are still controversial. By contrast, the discovery of MOTS-c may be a promising treatment or adjunctive therapy for postmenopausal women.

Interestingly, when MOTS-c was fused with cell penetrating peptide (PRR)_5_ to cross the blood–brain barrier, it enhanced the formation and consolidation of object and location recognition memory, while also ameliorating memory deficits induced by Aβ1-42 or LPS [91]. MOTS-c treatment significantly downregulated the expression of pro-inflammatory cytokines (including IL-6, IL-1β, TNF-α) in the hippocampus following LPS or Aβ1 treatment. However, MOTS-c treatment did not increase phosphorylation of ERK, JNK and p38 in these studies [91], which may indicate that the mechanisms of MOTS-c activity are tissue-specific.

#### MOTS-c in exercise and aging

Typical features of aging include a progressive decline of mitochondrial activity, reduced stress resilience, and worsening physical function [92]. As a peptide encoded by mitochondrial DNA, MOTS-c plays an active role in the regulation of stress, metabolism and maintenance of mitochondrial homeostasis. It can be speculated that MOTS-c has potential roles in promoting healthy aging, such as maintaining homeostasis of the body, improving physical function, and alleviating aging-related pathologies. Indeed, recent studies support this view. For example, MOTS-c significantly improved the physical function in mice of all ages. MOTS-c regulates the expression of genes related to metabolism and protein stabilization, skeletal muscle metabolism, and myocyte adaptation to stress [34, 58, 93]. Moreover, exercise promoted the expression of endogenous MOTS-c and increased the level of MOTS-c in skeletal muscle and plasma (returning to initial levels after 4 h of rest) [34]. However, the specific mechanism through which exercise regulates the expression of MOTS-c is not well understood. As described above, there appears to be positive feedback loop encompassing AMPK, PGC-1α and MOTS-c. Exercise increases MOTS-c expression via the AMPK-PGC-1α pathway, which in turn improves muscle homeostasis, increases exercise capacity, promotes glucose uptake, and improves stress resistance [57, 58]. In addition, exercise may also regulate MOTS-c production and secretion via lipocalin, an endogenous bioactive peptide secreted by adipocytes with insulin-sensitizing effects. Lipocalin was found to regulate the expression of MOTS-c in C2C12 myotubes by increasing the expression of SIRT1 and PGC-1α [56]. By contrast, it has been suggested that endurance exercise did not significantly reduce body weight or alter plasma MOTS-c levels in either healthy probands or polycystic ovarian syndrome patients [94]. Other studies found that exercise significantly increased MOTS-c levels after breast cancer surgery in non-Hispanic whites, but not in Hispanics [95]. This suggests that there may be ethnic or pathological differences in the increase in MOTS-c due to exercise. In addition, the mitochondrial DNA variant *m.1382A* > *C* leads to a K14Q amino acid substitution in MOTS-c and decreases the effect of MOTS-c on insulin sensitivity, thereby increasing the risk of T2D [96].

There is growing evidence that acute and long-term exercise can modulate endogenous MOTS-c levels, and that treatment of mice with MOTS-c improves exercise function. MOTS-c treatment significantly improved physical performance (significantly longer running time, increased endurance, increased maximal speed) in aged mice, in part by modulating skeletal muscle function and improving "metabolic flexibility". Furthermore, MOTS-c treatment completely reversed the preference of aged mice to utilize carbohydrates at night compared to middle-aged mice, showing a similar circadian rhythm pattern [34]. Body movement function is very important for healthy aging, and MOTS-c treatment of aged mice resulted in a trend towards increased median and maximum life span with decreased hazard ratios [34]. And in a study on muscle progenitor cell differentiation, MOTS-c enhanced myotube formation by reducing STAT3 transcriptional activity [97]. This suggests that MOTS-c may serve as a potential treatment for muscle atrophy. MOTS-c enhanced glycolytic flux and energy production in muscles affected Duchenne muscular dystrophy (DMD), in addition to improving muscle capacity in healthy mice [93]. MOTS-c promoted the uptake of the therapeutic agent phosphorodiamidate morpholino oligomer (PMO) in dystrophic muscles, increasing the abundance of dystrophin-positive muscle fibers, while also resulting in higher levels of dystrophin expression. Because MOTS-c may affect human muscle exercise capacity, doping control authorities have proposed a test to detect MOTS-c in plasma samples to prevent athletes using it as doping [98]. Although exercise can promote the expression of MOTS-c, the exact molecular mechanism is not clear. Regular moderate-intensity running strongly increased hypothalamic MOTS-c expression, whereas a single high-intensity run until exhaustion did not have this effect [10]. Moreover, hypothalamic production of ROS may be critical for these exercise-induced changes [10]. These findings emphasize the importance of exercise duration, mode and intensity for MOTS-c production. However, further experiments will be needed to clarify the mode of exercise, mechanism and tissue specificity that leads to the production of MOTS-c.

In addition to metabolic flexibility, insulin sensitivity and exercise function, MOTS-c improves cardiovascular function in aging mice, which is known to progressively deteriorate with age [46, 47, 99–102]. It was recently discovered that MOTS-c improves cardiovascular function through the AMPK pathway [102–105]. Similarly, MOTS-c treatment significantly reduced the number of disordered elastic fibers, decreased vascular calcification, and significantly improved vessel wall structure and vascular tone via the AMPK pathway [46]. In addition, AMPK activation upregulated the PI3K/AKT/eNOS pathway to protect coronary endothelium [99]. It was also recently proposed that MOTS-c may also improve endothelial dysfunction via the MAPK/NF-κB pathway, which is involved in endothelial dysfunction by regulating the surface expression of adhesion molecules such as VCAM-1 and ICAM-1 [100]. However, this hypothesis still lacks direct experimental proof. In the heart, MOTS-c improves myocardial mechanical efficiency, enhances cardiac systolic function, improves diastolic function, and reduces damage to heart structure and function from type 2 diabetes [47, 101, 102]. In addition, exogenous supplementation of MOTS-c promotes the cardiovascular benefits of exercise.

Overall, exercise enhances MOTS-c expression, thereby enhancing metabolic activity, insulin sensitivity, physical exercise function, and cardiovascular function through a variety of mechanisms. This allows MOTS-c to exhibit a powerful function in promoting healthy aging.

## Conclusions and challenges

Stress response or exercise can significantly activate MOTS-c expression and translocation to the nucleus to regulate the expression of multiple genes as a mitochondrial-nuclear retrograde signal. Activated MOTS-c mainly acts on the Folate-AICAR-AMPK pathway, thereby regulating energy metabolism, insulin resistance, the inflammatory response, brown adipose tissue activation, exercise, neuronal protection, aging and aging-related pathologies.

Although research on MOTS-c is gradually increasing, there are still many questions that remain unclear. For example, MOTS-c is translated in the cytoplasm, yet it is not clear how MOTS-c mRNA translocates from the mitochondria. It is also unclear how MOTS-c specifically regulates the expression of nuclear genes. MOTS-c may also interact with other organelles in addition to entering the nucleus, which also remains unknown. MOTS-c was previously thought to have no effect on cognitive function, but recently it has been found to facilitate the formation of object and location memory. MOTS-c is mainly found in skeletal muscle and plasma, but it acts differently in different tissues. Accordingly, the tissue specificity of MOTS-c action might be an interesting direction of future research. MOTS-c significantly alleviates the aging-related metabolic disorders, insulin resistance, cardiovascular impairment and reduced exercise function. Moreover, exercise stimulates the expression of MOTS-c, which gives it a strong potential for promoting healthy aging. However, different exercise intensities and durations in the general population or in obese and T2D patients can stimulate MOTS-c to produce different effects. It is therefore important to identify exercise modalities that will most effectively stimulate MOTS-c production. The clarification of these issues will facilitate the multiple beneficial effects of MOTS-c on metabolic homeostasis and healthy aging.

## Data Availability

Not applicable.
